# Investigation of an outbreak caused by an unknown infectious pathogen in rural Zola

**DOI:** 10.11604/pamj.supp.2021.40.2.30990

**Published:** 2021-12-16

**Authors:** Fortress Yayra Aku, Dinara Beisembekova, Nurudeen Ayobami Adebisi, Eva Mertens, Lea Wende, Sayed Mahdi Marashi, Janine Dywicki

**Affiliations:** 1 Department of Epidemiology and Biostatistics, School of Public Health, University of Health and Allied Sciences, Hohoe Campus, Ghana,; 2 Nazarbayev University, University Healthcare Department, Nur Sultan, Kazakhstan,; 3 Department of Chemical Pathology, University College Hospital, Ibadan, Oyo State, Nigeria,; 4 Bernhard Nocht Institute for Tropical Medicine, Department of Infectious Disease Epidemiology, Bernhard-Nocht-Strasse 74, 20359 Hamburg, Germany,; 5 Global Partnership Initiated Biosecurity Academia for Controlling Health Threats (GIBACHT), Hamburg, Germany,; 6 Robert Koch Institute, Centre for International Health Protection, Preparedness and Operations Support, Nordufer 20, 13353 Berlin, Germany,; 7 Global Partnership Initiated Biosecurity Academia for Controlling Health Threats (GIBACHT), Berlin, Germany,; 8 Tehran University of Medical Sciences, School of Public Health, Department of Virology, Tehran, Iran,

**Keywords:** Case study, biosafety, biosecurity, outbreak investigation, epidemiology, communication

## Abstract

This case study was written as part of a fellowship in biosafety and biosecurity organised by the German Biosecurity Programme, namely the Global Partnership Initiated Biosecurity Academia for Controlling Health Threats (GIBACHT). Among other objectives, the fellowship focuses on equipping participants with the skills of developing their own country-specific case studies with focus on biosafety- and biosecurity-related scenarios. Upon completion of the underlying case study, participants should be able to identify some existing gaps with regards to early detection and investigation of outbreaks, describe the key steps in outbreak investigation, explain the role of communication and coordination among the various stakeholders in outbreak investigations and analyse epidemiological data obtained during outbreak investigations. They should also be able to suggest appropriate control and prevention measures for specific disease outbreaks with focus on foodborne outbreaks and to distinguish between biosafety and biosecurity concepts.

## How to use this case study

### General instruction

This case study should be used as an add-on training and capacity-building resource for public health and epidemiology trainees meant to complement the theoretical aspects learned through lectures and/or other formats in a practical way. Also, it can serve as a supplementary material in the context of in-service training activities for frontline public health professionals to refresh their knowledge and skills regarding the principles and concept of outbreak investigation and related biosafety as well as biosecurity matters. Ideally, the case study should be led by 2 facilitators per group of around 5 participants each. To enhance interactivity, participants should read the subsequent paragraphs of the case study in turns. Discussions within the group during the conduction of the case study constitute a key element of the process and should be oriented towards the collective identification of an outbreak investigation strategy as a team. Respectively, facilitators should lead the discussion through guiding questions and further hints making use of materials such as flip charts. The integration of role plays as a facilitation method can also enhance the overall interactivity of the case study at hand.

### Audience

The underlying case study targets a broad range of public health professionals working in outbreak prevention and control, especially disease control and surveillance officers, students of epidemiology training programs and other public health practitioners.

### Prerequisites

Prior to the use of this case study, participants should have basic background knowledge of the principles of outbreak investigation as well as biosafety and biosecurity issues related to the handling of samples in the context of outbreak investigations.

### Materials required

Computer with MS Excel or other spreadsheet software, pen, notebooks, calculator, flip charts and markers.

### Level of training and associated public health activity

Intermediate level in outbreak investigation

### Time required

This case study is expected to take between 2.5 to 3 hours.

### Language

English

## Case study material

- Download the case study student guide (PDF - 561 KB)
- Request the case study facilitator guide: contact info@gibacht.org
